# Alterations of Gene Expression and Signaling Pathway Activity in Venous Smooth Muscle Cells After Uremic Serum Exposure

**DOI:** 10.3390/ijms27188148

**Published:** 2026-09-13

**Authors:** Youyou Zheng, Unimunkh Uriyanghai, Christine Wai, Mihaela Mocanu, John S. Poulton, Prabir Roy-Chaudhury, Gang Xi

**Affiliations:** 1 UNC Kidney Center, Division of Nephrology and Hypertension, Department of Medicine, The University of North Carolina at Chapel Hill, Chapel Hill, NC 27599, USA; yoyoz@email.unc.edu (Y.Z.); unimunckh_uriyanghai@med.unc.edu (U.U.); cwai@email.unc.edu (C.W.); mihaela_mocanu@med.unc.edu (M.M.); poultonj@email.unc.edu (J.S.P.); prabir_roy-chaudhury@med.unc.edu (P.R.-C.); 2 WG (Bill) Hefner VA Medical Center, Salisbury, NC 28144, USA

**Keywords:** venous smooth muscle cells, uremia, bulk RNA sequencing, venous thrombosis, venous stenosis

## Abstract

Uremia is the most common pathophysiological symptom in chronic kidney disease (CKD) patients, particularly in end-stage kidney disease (ESKD) patients. Uremia can lead to several vein-specific vascular diseases, such as renal vein thrombosis (RVT), deep vein thrombosis (DVT) and dialysis access-induced venous stenosis. One of our previous studies demonstrated that uremic serum exposure induced different cellular responses in pig venous smooth muscle cells (vSMCs) compared with arterial smooth muscle cells (aSMCs). To explore the underlying mechanisms responsible for vein-specific cellular responses, bulk RNA sequencing was utilized to examine differential gene expression in porcine vSMCs after uremic serum exposure. Differentially expressed genes (DEG) analysis revealed that 408 genes were upregulated, and 387 genes were downregulated after uremic serum treatment. Gene Ontology Biological Process analysis demonstrated that uremic serum exposure led to transcriptomic downregulation of cellular energy expenditure activities, such as the Cell Cycle, and positive transcriptomic enrichment of Cellular Response to Endoplasmic Reticulum (ER) stress, unfolded protein response and hypoxia. Both Gene Set Enrichment Analysis (GSEA) and Overrepresentation Analysis (ORA) obtained similar results. ORA revealed additional signaling pathways predicted to be transcriptomically downregulated, such as the Hippo signaling pathway and Focal Adhesion and Cytoskeletal Structure Regulating pathways. ORA also predicted several positively enriched signaling pathways related to cellular stress responses and waste disposal. To precisely identify vSMCs-specific alterations, an interaction-based KEGG GSEA was performed. The analysis revealed several significantly different responses between vSMCs and aSMCs, such as Protein Processing in the Endoplasmic Reticulum, Integrated Stress Response signaling pathway and Mitophagy, which showed more positive responses in vSMCs, while Oxidative Phosphorylation showed a more positive response in aSMCs. These altered signaling pathways may be responsible for vein-specific clinical symptoms, such as venous segment stenosis in arteriovenous fistula, observed in CKD/ESKD patients.

## 1. Introduction

Chronic kidney disease (CKD) is one of the most common and fastest-growing modern diseases, causing significant public health burden worldwide [1]. As kidney function declines, patients become increasingly susceptible to systemic complications, such as vascular diseases [2]. In patients with advanced CKD and end-stage kidney disease (ESKD), cardiovascular and vascular complications are major contributors to morbidity and mortality [3]. One of the central metabolic abnormalities in CKD is uremia, a condition characterized by the retention of urea and other toxins that are normally cleared by the kidneys [4]. Uremia has broad effects on the vasculature, promoting inflammation, oxidative stress, endothelial injury, vascular calcification, and abnormal remodeling of vascular smooth muscle cells (VSMCs) [5,6]. Although arterial complications of CKD have been widely studied, venous vascular disease is also highly relevant, particularly in the setting of dialysis access and CKD-associated thrombotic complications [7].

Veins are biologically and clinically distinct from arteries. Under normal anatomical conditions, classic atherosclerotic lesions occur mainly in arteries and are rarely observed in veins [8]. However, veins are highly susceptible to stenosis, thrombosis, fibrosis, and maladaptive remodeling after vascular injury or intervention, including arteriovenous fistula (AVF) creation, balloon angioplasty, and stent placement [9,10]. This distinction is especially important in patients receiving hemodialysis, for whom AVFs are commonly created to provide long-term vascular access. In AVF veins, high-pressure arterial blood flows directly into the venous system, exposing the vein wall to elevated pressure, altered shear stress, and repeated puncture injury [11]. These abnormal conditions can trigger structural and functional complications, including aneurysm formation, stenosis, thrombosis, venous hypertension, and access dysfunction [12]. In a cross-sectional study of patients undergoing hemodialysis through native AVFs, aneurysmal changes were identified in 60% of patients and were associated with longer dialysis duration and higher flow dialysis exposure [13]. Repeated cannulation and altered hemodynamic stress may also contribute to vascular dilation and structural changes at AVF puncture sites [14].

At the cellular level, VSMCs play a central role in maintaining vessel wall integrity and regulating vascular tone. However, under pathological conditions, VSMCs can undergo marked phenotypic and functional changes [15]. Previous studies have shown that venous smooth muscle cells (vSMCs) and arterial smooth muscle cells (aSMCs) differ in important ways in different environmental settings, disease conditions, and injury responses [9,16]. Our previous in vitro studies demonstrated that, compared to aSMCs, vSMCs have higher proliferation and migration, greater susceptibility to dedifferentiation cues such as platelet-derived growth factor-BB (PDGF-BB), altered expression of focal adhesion and extracellular matrix-related proteins, and stronger activation of signaling pathways including JNK, p38MAPK, and AKT in vSMCs [16]. Previous transcriptomic studies, including microarray hybridization, bulk RNA sequencing, and single-cell RNA sequencing, have also revealed important artery–vein differences in gene expression related to contractile phenotype, extracellular matrix composition, fibroblast activation, inflammatory potential, and response to oxidative stress [8,17]. Importantly, our bulk RNA-seq studies clearly demonstrated that vSMCs and aSMCs use different sets of genes to regulate the same pathway [18]. In addition, aSMCs exposed to uremic serum displayed transcriptomic enrichment in signaling pathways that regulate cellular redox homeostasis, the cellular waste disposal system and ER stress [19]. These findings indicate that veins and arteries are not simply passive conduits but have distinct biological programs that may affect their responses to uremic injury.

Despite these insights, the effects of uremic conditions specifically on vSMCs remain unclear. Many previous in vitro studies relied on candidate-gene approaches. For example, one of our prior studies demonstrated that uremic serum-stimulated cell migration was more potent in vSMCs, compared to aSMCs, whereas uremic serum-stimulated expression of early osteogenic markers and inflammatory responses were more potent in aSMCs compared to vSMCs [20]. However, those targeted investigations overlook broader transcriptomic changes and additional signaling pathways not specifically examined. In addition, previous sequencing studies have often focused on baseline artery–vein differences, organ-specific vascular heterogeneity, or tissue-level cell composition rather than the specific effects of uremic serum on vSMCs [17]. Similar limitations exist in uremia studies, in which blood cells or calcified vascular tissues were examined, but no transcriptomic studies have investigated the effect of uremic toxins on vSMCs [21,22]. Therefore, a comprehensive and unbiased approach is needed to define how vSMCs respond to the uremic environment.

In this study, bulk RNA sequencing was used to investigate the transcriptomic response of porcine vSMCs to uremic serum. Porcine cells were used because pigs are anatomically and physiologically more similar to humans than small rodent models. This study aims to provide new insight into the molecular basis of CKD/uremia-related venous diseases and venous remodeling by identifying differentially expressed genes (DEGs) and transcriptomically enriched signaling pathways in vSMCs after uremic serum exposure. Alterations in gene expression under uremic conditions may reveal some of the molecular mechanisms driving venous remodeling in CKD-associated venous diseases, such as AVF stenosis, thrombosis, and access failure, which remain major clinical challenges for hemodialysis patients.

## 2. Results

### 2.1. Uremic Serum Alters Global Gene Expression Profiles in vSMCs

**Figure 1:**
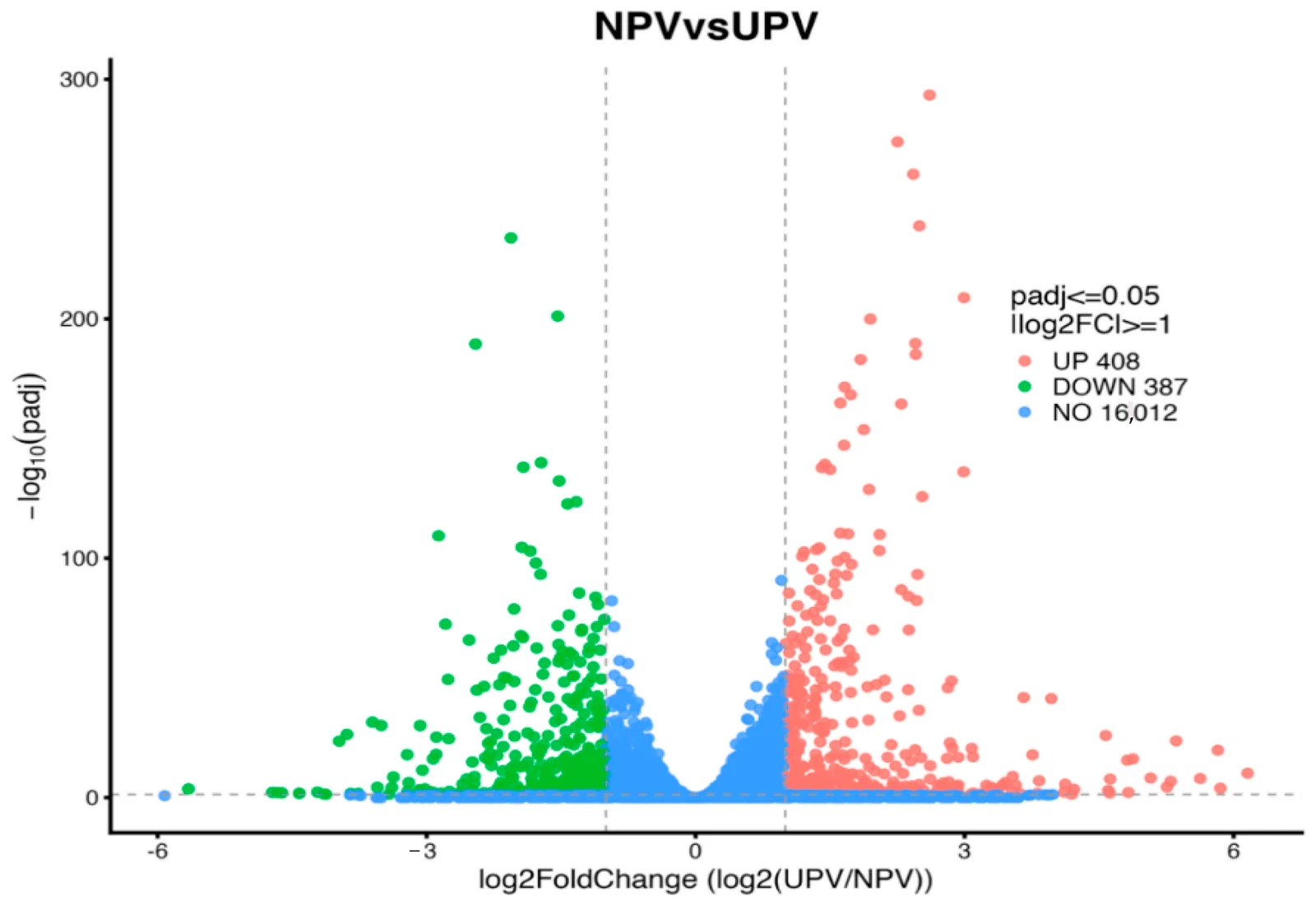
Global transcriptomic differences between normal porcine serum-treated venous smooth muscle cells (NPV) and uremic porcine serum-treated venous smooth muscle cells (UPV). Volcano plot of all tested genes showing log2FC (UPV/NPV) versus −log10(padj). Dashed vertical lines indicate the fold-change cutoff of |log2FC| ≥ 1, and the dashed horizontal line indicates the significance cutoff of padj ≤ 0.05. Red points indicate genes significantly upregulated in UPV; green points indicate genes significantly downregulated in UPV; blue points indicate genes without significant differential expression.

To investigate the transcriptomic response of primary porcine vSMCs to uremic conditions, bulk RNA sequencing was performed on vSMCs treated with normal porcine serum (NPV) or uremic porcine serum (UPV). Differential expression analysis was conducted using log2FC calculated as log2 (UPV/NPV); thus, genes with positive log2FC values were more highly expressed in UPV, while genes with negative values were downregulated in UPV. The volcano plot demonstrated clear transcriptomic changes after uremic serum exposure (Figure 1). Using the cutoff of padj ≤ 0.05 and |log2FC| ≥ 1, we found that 408 genes were significantly upregulated and 387 genes were significantly downregulated in UPV compared with NPV. A total of 16,012 genes did not meet the significance and fold-change thresholds. These findings indicate that uremic serum exposure induces broad but selective transcriptomic remodeling in vSMCs (Figure 1).

### 2.2. Functional Enrichment Analysis of vSMCs After Uremic Serum Exposure

**Figure 2:**
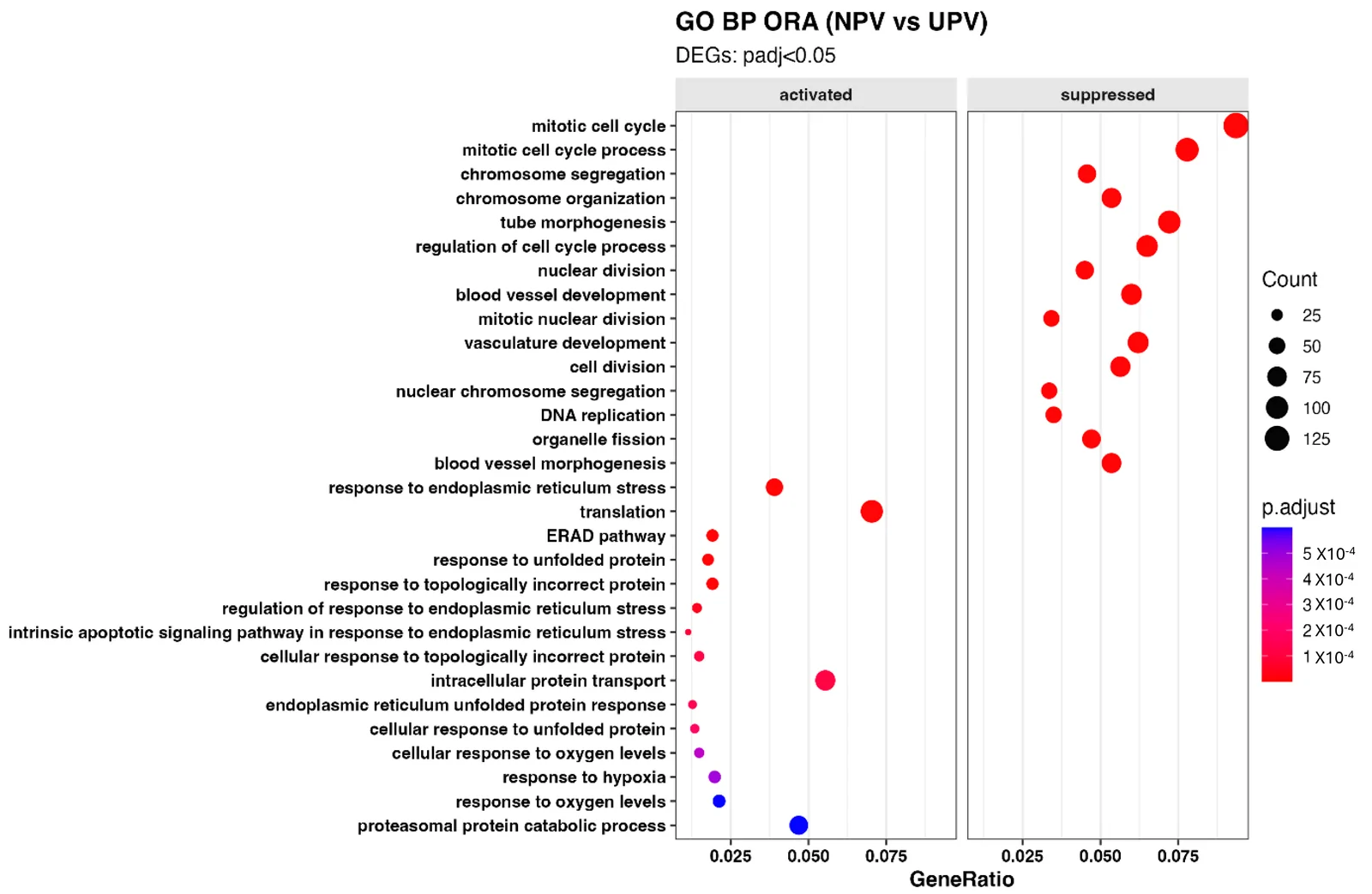
Gene Ontology biological process enrichment in vSMCs after exposure to uremic porcine serum. Dot plot of enriched GO biological process (BP) terms for differentially expressed genes between NPV and UPV. DEGs were defined using padj < 0.05 and were separated by direction of expression change. The left panel shows biological processes enriched among genes upregulated in UPV, labeled as activated, and the right panel shows biological processes enriched among genes downregulated in UPV, labeled as suppressed. The *x*-axis shows GeneRatio, representing the fraction of input genes annotated to each GO term. Dot size indicates the number of genes mapping to each term, and dot color indicates adjusted *p*-value.

Gene ontology (GO) biological process (BP) overrepresentation analysis (ORA) revealed distinct functional patterns between genes upregulated and downregulated in UPV. Among the biological processes associated with downregulated genes, the most prominent terms were related to Cell Cycle progression and chromosome dynamics, including Mitotic Cell Cycle, Chromosome Segregation, Cell Division, DNA Replication, and Organelle Fission. In addition to Cell Cycle pathways, several vascular development-related processes were also enriched for downregulated genes, including Vasculature Development and Blood Vessel Morphogenesis (Figure 2).

In contrast, biological processes that were predicted to be positively enriched signaling pathways include stress and protein-homeostasis responses, such as Responses to Endoplasmic Reticulum (ER) stress, ER-associated degradation pathway, response to Unfolded Protein, and Intracellular Protein Transport. Several oxygen-related response terms were also enriched, including response to hypoxia and cellular response to oxygen levels. Together, these findings suggest that vSMCs exposed to uremic serum may upregulate genes that are associated with ER stress, unfolded protein response (UPR), proteasomal degradation, and oxygen-stress-related transcriptomic programs (Figure 2).

**Figure 3:**
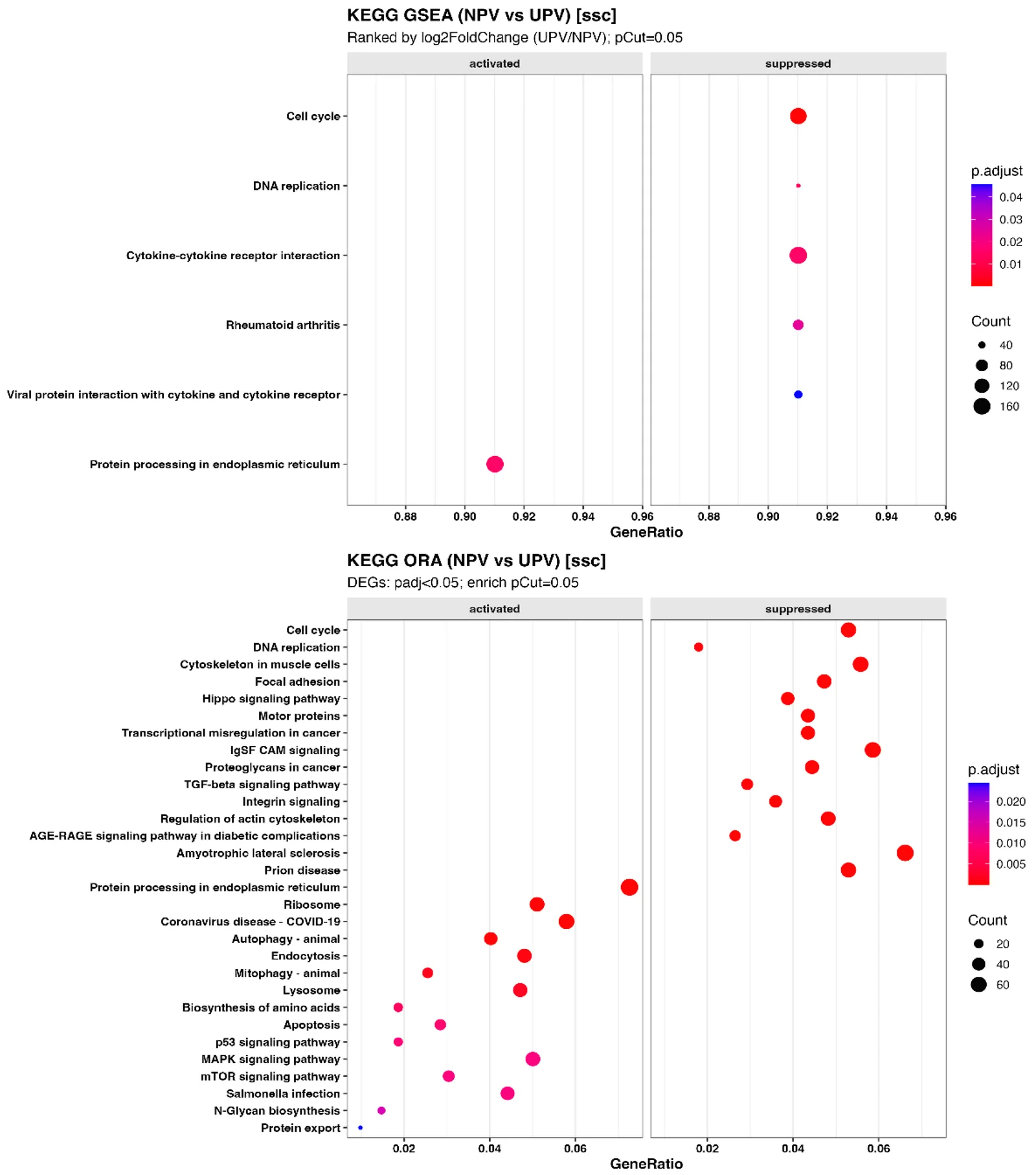
**KEGG pathway enrichment by GSEA and ORA in NPV vs. UPV.** KEGG pathway enrichment analysis was performed using both gene set enrichment analysis (GSEA) and overrepresentation analysis (ORA). For GSEA, all genes were ranked by log2FC (UPV/NPV), and pathways were separated into activated or suppressed groups based on enrichment direction in UPV relative to NPV. For ORA, DEGs were defined using padj < 0.05 and analyzed separately by direction of expression change. In both plots, the *x*-axis shows GeneRatio, dot size indicates the number of genes contributing to each pathway, and dot color indicates adjusted *p*-value.

KEGG pathway analysis was performed using both Gene Set Enrichment Analysis (GSEA) and ORA to identify pathway-level changes induced by uremic serum exposure. GSEA showed positive enrichment of protein processing in the ER in UPV, consistent with the GO enrichment results. In contrast, several pathways were predicted to be transcriptomically downregulated in UPV, including Cell Cycle, DNA Replication, and Cytokine–Cytokine Receptor Interaction (Figure 3 top). The transcriptomic downregulation of Cell Cycle and DNA Replication pathways is consistent with the GO BP analysis results showing decreased expression of genes associated with mitotic and chromosomal programs.

KEGG ORA provided additional pathway-level resolution. Pathways that were predicted to be transcriptomically downregulated were Cell Cycle, DNA Replication, Cytoskeleton in Muscle Cells, Focal Adhesion, Hippo signaling pathway, Motor Proteins, TGF-β signaling pathway, Integrin signaling, Regulation of Actin Cytoskeleton, and AGE-RAGE signaling pathway in diabetic complications (Figure 3 bottom). Pathways enriched among genes upregulated in UPV included Protein Processing in the Endoplasmic Reticulum, Autophagy-Animal, Endocytosis, Mitophagy-animal, Lysosome, Biosynthesis of Amino Acids, Apoptosis, P53 signaling pathway, MAPK signaling pathway, and mTOR signaling pathway (Figure 3 bottom). Enrichment of these pathways among upregulated genes suggests that the transcriptomic response of vSMCs to uremic conditions involves stress adaptation, protein processing, autophagy, endocytosis, lysosomal functions, Apoptosis-associated signaling, and intracellular trafficking.

### 2.3. KEGG Pathways Mapping of DEGs

To explore the specific DEGs in the context of a specific pathway, DEGs were projected onto the relevant KEGG pathways that were detected in GO BP ORA (NPV vs. UPV) analysis (Figure 2), and KEGG GSEA (NPV vs. UPV) and KEGG ORA (NPV vs. UPV) (Figure 3). Gene-level log_2_FC values from DEGs were projected onto pathway diagrams using the Pathview (version 1.50.0) KEGG database [23,24].

#### 2.3.1. Uremic Serum Exposure Alters Signaling Pathways That Regulate Cell Cycle and Cell Proliferation

**Figure 4:**
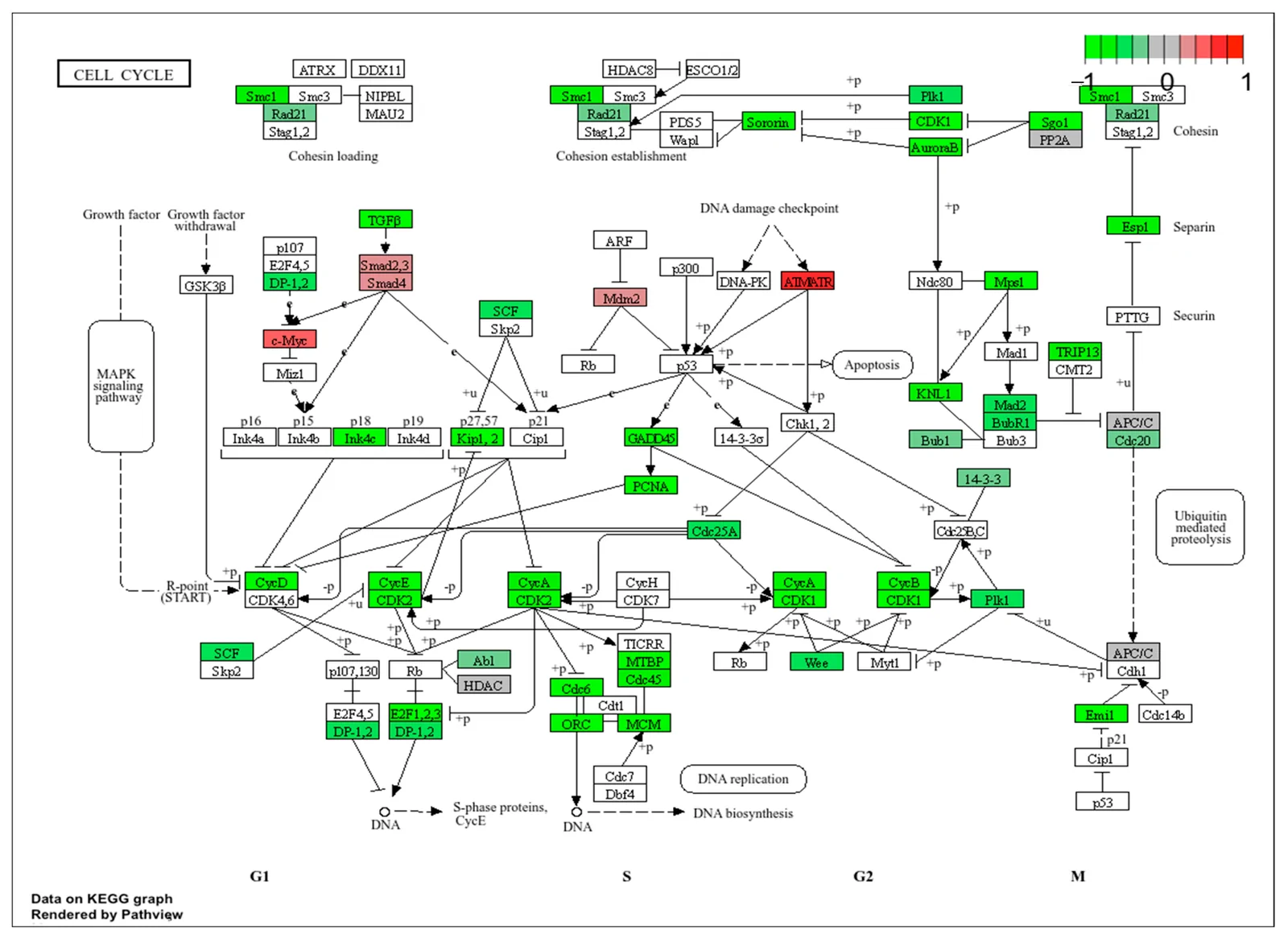
**Cell Cycle pathway map in vSMCs exposed to uremic serum.** KEGG Cell Cycle pathway with DEG log2FC values overlaid using Pathview. Node color represents log2FC (UPV/NPV), with red indicating upregulation in UPV and green indicating downregulation in UPV relative to NPV. The pathway structure is based on the KEGG pathway database (https://www.kegg.jp/kegg/pathway.html) (accessed on 14 April 2026).

Both KEGG GSEA and ORA analyses revealed that Cell Cycle and DNA Replication were predicted to be transcriptomically downregulated in vSMCs after uremic serum exposure. When DEGs in the Cell Cycle pathway were examined, a large group of genes was found to be downregulated, including *Smc1*, *TGFB*, *Ink4c*, *Kip1/2*, *CycD*, *CycE*, *CDK2*, *CycA*, *E2F1/2/3*, *GADD45*, *PCNA*, *Cdc6*, *ORC*, *MTBP*, *MCM*, *Sororin*, *CDK1*, *AuroraB*, *Mps1*, *KNL1*, *Sgo1*, *Esp1*, and *Emi1* (Figure 4). It is worth noting that some of these downregulated genes are suppressors of the Cell Cycle, such as *Kip1/2*, *Ink4c* and *GADD45*. We also detected upregulation of a few genes in this pathway. While the net effect of these changes on the Cell Cycle is difficult to decipher, the observation that so many genes in this pathway were altered suggests significant perturbation of the Cell Cycle in cultured vSMCs after exposure to uremic serum.

**Figure 5:**
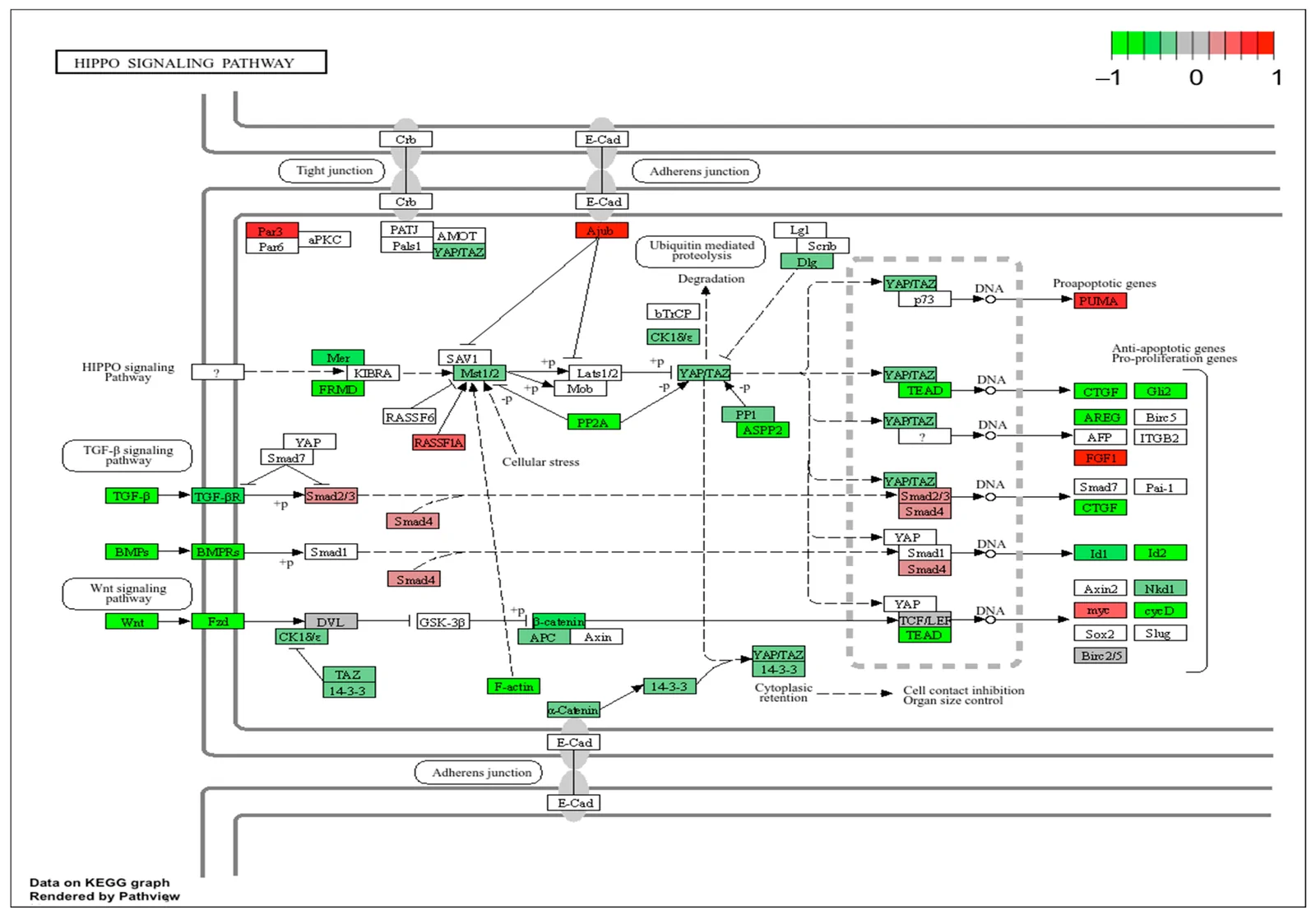
**Hippo signaling pathway map in vSMCs exposed to uremic serum.** KEGG Hippo signaling pathway with DEG log2FC values overlaid using Pathview. Node color represents log2FC (UPV/NPV), with red indicating upregulation in UPV and green indicating downregulation in UPV. The pathway structure is based on the KEGG pathway database (https://www.kegg.jp/kegg/pathway.html) (accessed on 14 April 2026).

KEGG ORA also revealed that the Hippo signaling pathway, which normally inhibits cell proliferation, was predicted to be transcriptomically downregulated in vSMCs after uremic serum exposure. A large group of genes in this pathway were downregulated in UPV, including *TGFB*, *BMPs*, *Wnt*, *Fzd*, *FRMD*, *F-actin*, *PP2A*, *ASPP2*, *TEAD*, *CTFG, Gli2*, *AREG*, *Id2* and *CycD*, while only Par3, Ajub, PUMA and FGF1 were upregulated (Figure 5). In addition, KEGG ORA revealed that the P53 signaling pathway, MAPK signaling pathway and mTOR signaling pathway were predicted to be positively enriched in vSMCs after uremic serum exposure (Figure 3). These pathways are very important to regulate cells proliferation in vivo.

#### 2.3.2. Uremic Serum Exposure in vSMCs Upregulates Genes Associated with Cellular Stress Response and Waste-Disposal Programs

**Figure 6:**
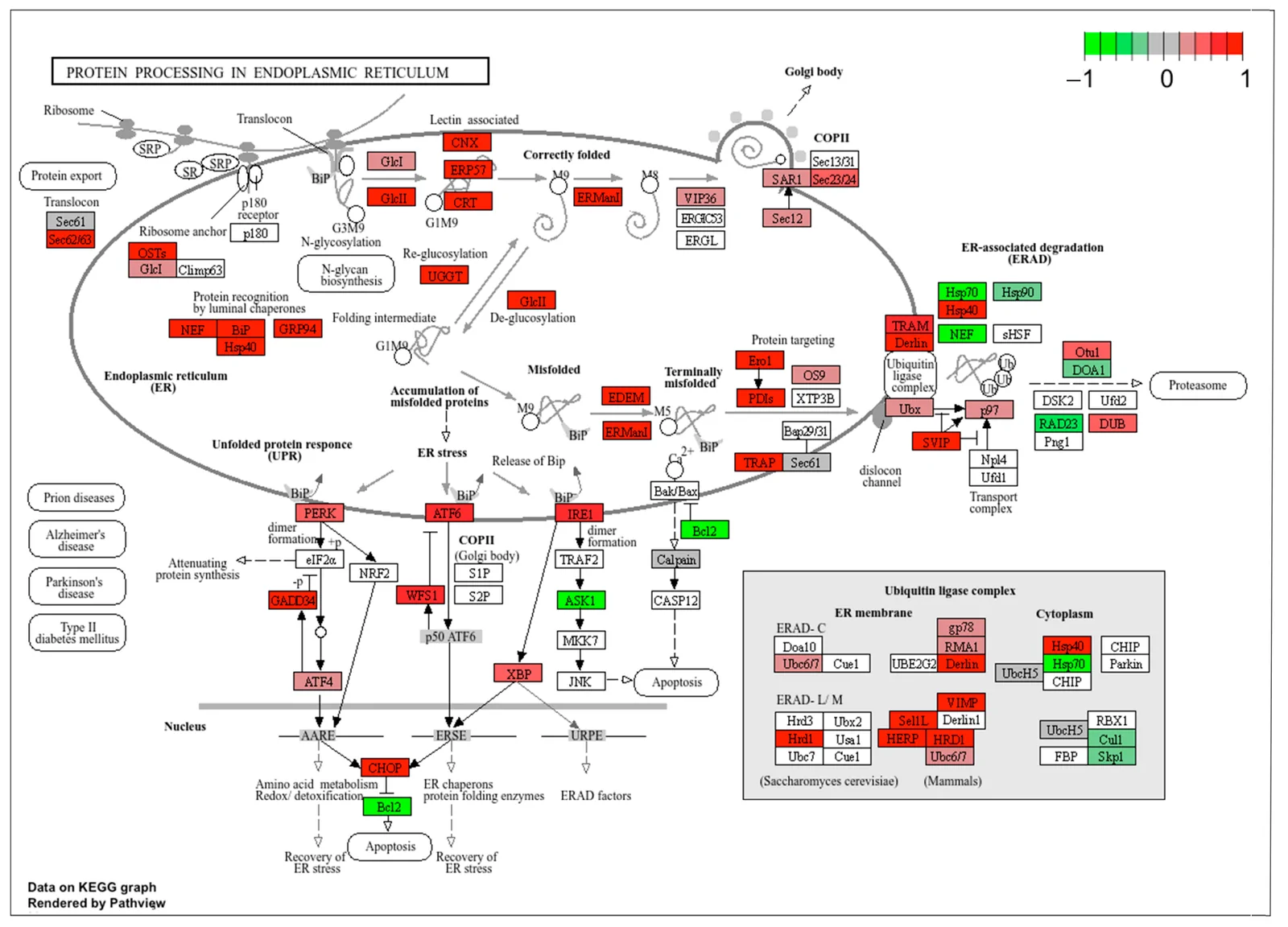
**Protein Processing in Endoplasmic Reticulum (ER) signaling pathway in vSMCs after uremic serum exposure.** KEGG protein processing in the ER signaling pathway with DEG log2FC values overlaid using Pathview. Node color represents log2FC (UPV/NPV), with red indicating upregulation in UPV and green indicating downregulation in UPV. This map highlights altered expression of genes involved in UPR, ER-associated degradation, and ubiquitin ligase complexes. The pathway structure is based on the KEGG pathway database (https://www.kegg.jp/kegg/pathway.html) (accessed on 14 April 2026).

Functional analysis revealed that multiple signal pathways that regulate cellular stress responses were predicted to be positively enriched after uremic serum exposure, such as Response to ER Stress, ER UPR, and Cellular Waste-Disposal programs (e.g., Autophagy, Mitophagy and Lysosomes). When DEGs were projected onto the Protein Processing in ER pathway, a large group of gene expressions was upregulated, including *Sec62/63*, *OSTs*, *NEF*, *BiP*, *GRP94*, *Hsp40*, *GlcII CNX*, *ERP57*, *CRT*, *UGGT*, *ERManI*, *EDEM*, *Ero1*, *PDIs*, *TRAP*, *TRAM*, *Derlin*, *ATF6*, *IRE1*, *Hsp40*, *SVIP*, *GADD34*, *WFS1* and *CHOP* (Figure 6).

**Figure 7:**
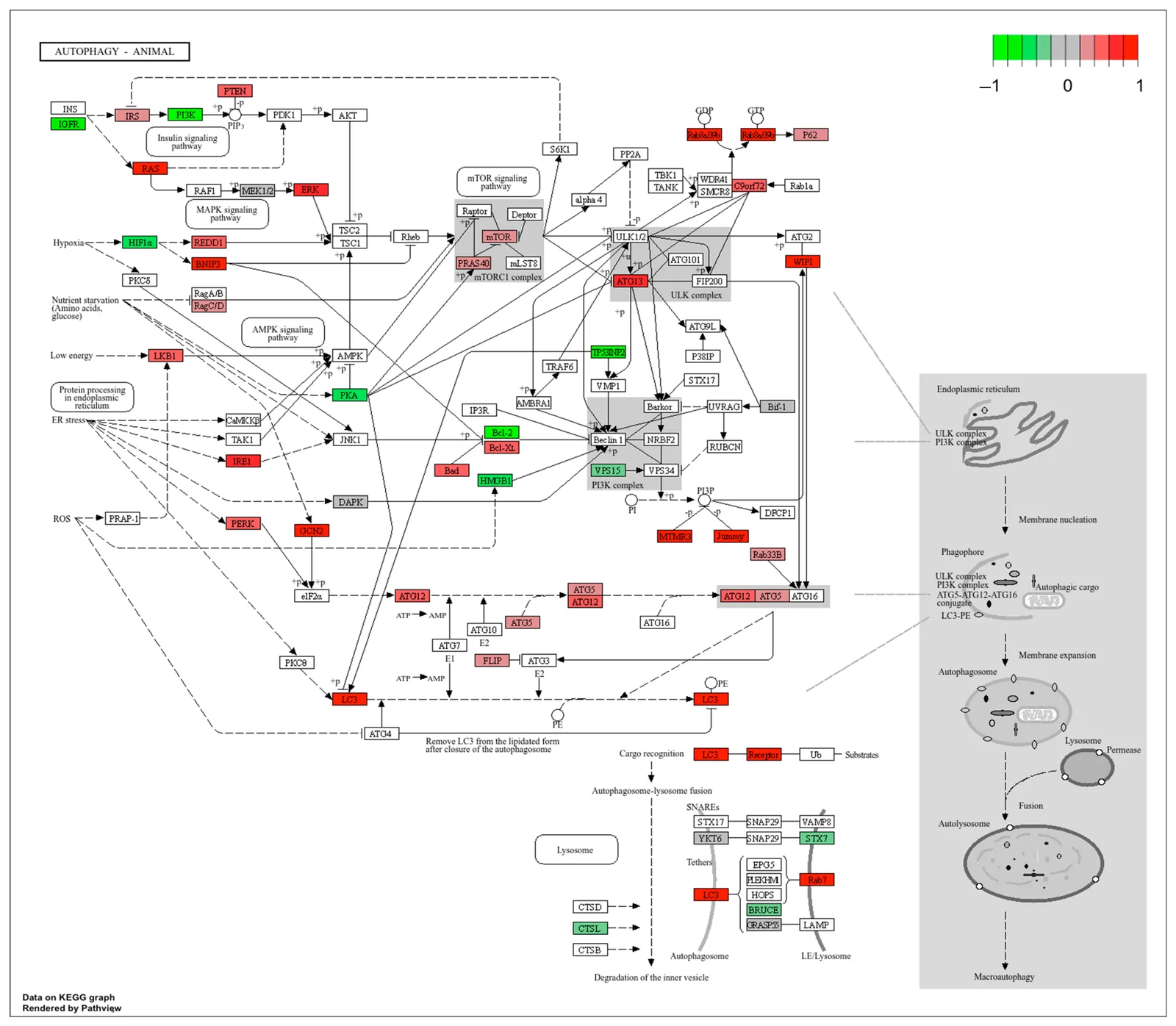
**Autophagy pathway map in vSMCs exposed to uremic serum.** KEGG autophagy-animal pathway with DEG log2FC values overlaid using Pathview. Node color represents log2FC (UPV/NPV), with red indicating upregulation in UPV and green indicating downregulation in UPV relative to NPV. This map highlights altered expressions of genes involved in autophagosome formation, ER stress-related autophagy regulation, lysosomal fusion, and cellular waste-disposal pathways. The pathway structure is based on the KEGG pathway database (https://www.kegg.jp/kegg/pathway.html) (accessed on 14 April 2026).

In the autophagy pathway, a large group of genes was upregulated after uremic serum exposure, including *RAS*, *ERK*, *BNIP3*, *IRE1*, *GCN2*, *LC3*, *ATG13*, *Rab8a/39b*, *WIPI*, *MTMR3*, *Jummy*, *Receptor* and *Rab7* (Figure 7). Meanwhile, in the Mitophagy pathway, the expression of *RAS*, *BNIP3*, *TFEB*, *Rab7* and *LC3* was also upregulated (Supplemental Figure S1).

Together, these results indicate that vSMCs exposed to uremic serum may upregulate genes that are associated with cellular stress adaptation pathways, including autophagy, ER stress, lysosomal activity, and Mitophagy. These responses may represent compensatory mechanisms to manage damaged proteins, altered organelles, and metabolic stress under uremic conditions.

#### 2.3.3. Uremic Serum Exposure Alters vSMCs Contractility, Cytoskeleton Structure and Phenotypical Switch

The Cytoskeleton in muscle cells pathway map revealed extensive changes in extracellular matrix, membrane junction, and nuclear envelope-associated genes. A large group of genes associated with extracellular matrix and membrane–cytoskeletal structure was downregulated after uremic serum exposure, including *COL*, *VCAN*, *ELM*, *THBS*, *ITGA*, *ITGB*, *DES*, *ACTB/G*, *SGCD*, *ATP1A/B*, *MYH*, *MYBPH*, *TPM1*, *TPM2*, *MYOM*, *PDLIM*, *FHL*, *ANKRD*, *TMOD*, and *MYOM*. Conversely, genes such as *COL6A*, *FBLN1_2*, *SGCB*, *VIM*, *TNNI1*, *TNNI3*, *NEBL*, *MYOZ*, *ANKB*, and *SYNE1,* were upregulated (Supplemental Figure S2).

KEGG ORA also demonstrated that the Focal Adhesion pathway was predicted to be transcriptomically downregulated after uremic serum exposure in vSMCs. The pathway map showed broad downregulation of genes involved in extracellular matrix interaction, Integrin signaling, and actin organization, including *ITGA/ITGB*, *caveolin*, *RTK*, *Shc*, *PI3K*, *actinin*, *filamin*, *vinculin*, *parvin*, *MLCP*, *MLCK*, *actin*, *β-catenin*, *Cyclin D* and *Bcl-2*. In contrast, expression of *ECM*, *PAK*, and *ERK1/2* was increased in this pathway (Supplemental Figure S3). These changes suggest that uremic serum may disrupt cell adhesion and cytoskeletal dynamics in vSMCs.

Furthermore, KEGG ORA demonstrated that the TGF-β signaling pathway was predicted to be transcriptomically downregulated in vSMCs after uremic serum exposure (Figure 3). When DEGs were projected onto the KEGG map, a large group of genes was downregulated, including *Activin B*, *BMPR1*, *RGMa/b*, *GERM*, *BMP*, *THBS1*, *THSD4*, *TGFβ*, *TGFβRII*, *FST*, *Lefty*, *GARP* and *Id*. Meanwhile, several other genes were upregulated, including *BMP2/4/6*, *Tfr1*, *Activin*, *ERK*, and *TMEM53* (Supplemental Figure S4).

These results may be especially relevant to venous remodeling because vSMCs adhesion, migration, and extracellular matrix interaction are central to stenosis, neointimal hyperplasia, and AVF dysfunction. These results suggest that uremic serum exposure may affect cytoskeletal and contractile-associated gene programs in vSMCs. The mixed regulation of contractile, extracellular matrix, and nuclear-cytoskeletal genes may reflect a shift in vSMCs phenotype under uremic stress.

#### 2.3.4. Uremic Serum Exposure Upregulates Genes Associated with Hypoxic Response in vSMCs

**Figure 8:**
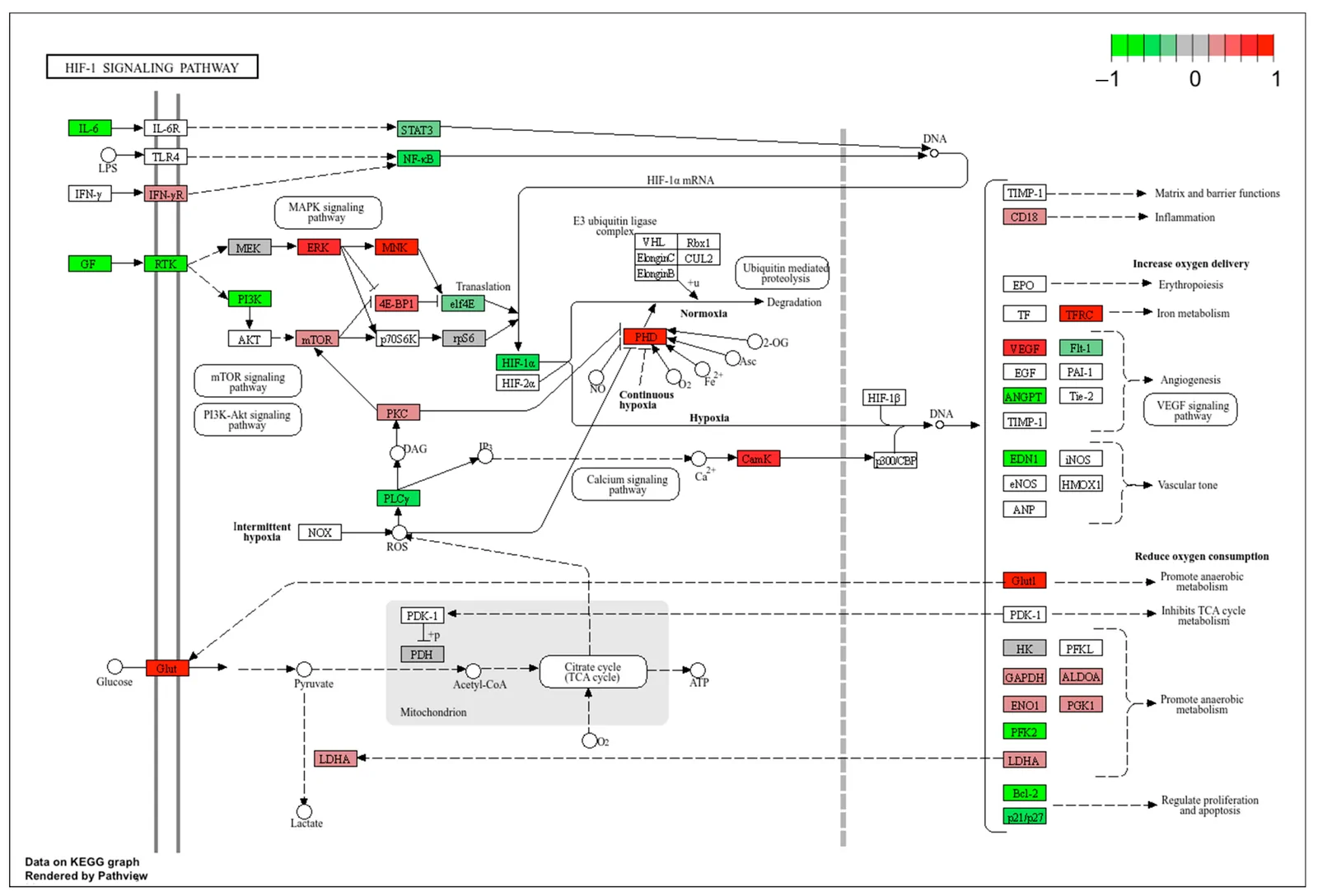
**HIF-1 signaling pathway map in vSMCs exposed to uremic serum.** KEGG HIF-1 signaling pathway with DEG log2FC values overlaid using Pathview. Red nodes indicate genes upregulated in UPV relative to NPV, and green nodes indicate genes downregulated in UPV. The pathway structure is based on the KEGG pathway database (https://www.kegg.jp/kegg/pathway.html) (accessed on 14 April 2026).

Another important change induced by uremic serum exposure in vSMCs is the upregulation of genes associated with Cellular Response to Oxygen Levels and Response to Hypoxia. The hypoxia-inducible factor (HIF) pathway is the master oxygen-sensing system in cells. We therefore projected DEGs onto this pathway, revealing upregulation of *Glut*, *ERK*, *MNK*, *PHD*, *CamK*, *TFRC*, *VEGF* and *Glut1*, and downregulation of *GF*, *IL-6*, *RTK*, *PI3K*, *HIF-1α*, *PLCγ*, *NF-κB*, *ANGPT*, *END1*, *PFK2* and *Bcl-2* after uremic serum exposure (Figure 8).

#### 2.3.5. Direct Comparison of Transcriptomic Data Between vSMCs and aSMCs

To compare the current vSMCs transcriptomic data with our parallel study on aSMCs, we used a cell type vs. serum type interaction analysis to directly compare gene-level effect sizes and magnitude of pathway enrichment. This analysis demonstrated that, at the gene level, 5096 genes showed a significant interaction at FDR < 0.05, and 3813 remained significant at FDR < 0.01. The arterial and venous uremic-serum effect sizes also showed almost no overall correlation (Pearson r = −0.01), suggesting that the transcriptomic responses differ substantially between the two subtypes of cells (Supplemental Figure S5). The interaction-based KEGG GSEA also identified several pathways with significantly different responses. In particular, Protein Processing in the Endoplasmic Reticulum, Integrated Stress Response signaling pathway and Mitophagy showed much more positive responses in vSMCs, while Oxidative Phosphorylation showed a more positive response in aSMCs (Supplemental Figure S6).

## 3. Discussion

Bulk RNA sequencing revealed that uremic serum exposure induces broad transcriptomic remodeling in porcine vSMCs. A total of 408 genes were significantly upregulated and 387 genes were significantly downregulated in UPV compared with NPV. Functional enrichment analyses showed the transcriptomic downregulation of Cell Cycle, DNA Replication, Chromosome Organization, Focal Adhesion, Integrin signaling, Actin Cytoskeleton Regulation, and Cytoskeleton in Muscle Cells signaling pathways. In contrast, uremic serum induced upregulation of many genes associated with several key pathways such as ER stress, Autophagy, Mitophagy, Lysosome, Apoptosis, Hypoxia, MAPK, p53 and mTOR signaling pathways. These findings suggest that vSMCs exposed to uremic serum may shift away from differentiated and structural maintenance programs toward stress-response and cellular waste-disposal pathways. Though future studies will be required to determine the effects of these changes in gene expression on vSMCs behavior, the transcriptomic changes in the pathways we have identified may contribute to CKD- and ESKD-associated venous remodeling, including AVF stenosis, thrombosis, and access dysfunction.

In a recent study in aSMCs exposed to uremic serum [19], we observed enrichment of DEGs associated with the inhibition of cellular energy expenditure, including processes and pathways such as Cell Cycle, DNA Replication and Cell Division. We detected these same transcriptomic patterns in vSMCs after uremic serum exposure. These changes can be part of a normal cellular response to stressful environments as the cells attempt to save energy and maintain survival. Interestingly, the Hippo signaling pathway, which normally negatively regulates cell proliferation, was also predicted to be transcriptomically downregulated after uremic serum exposure. Furthermore, KEGG pathway analysis showed that Cell Cycle negatively regulating genes, such as *Kip1/2*, *Ink4c* and *GADD45*, were downregulated. Meanwhile, in contrast with our findings in aSMCs, MAPK and mTOR signaling pathways were predicted to be positively enriched in vSMCs after uremic serum exposure. Therefore, the enhanced cell proliferation after uremic serum exposure in vSMCs that we previously observed [20] may be due to the combined effects of these changes. However, it is important to note that the current study only describes the transcriptomic changes after uremic serum exposure. The biological consequences of these changes are determined by many downstream processes, such as translational efficiency, post-translational modification and the integrated effects of changes in multiple signaling pathways. In addition, we detected expression changes in genes that negatively regulate a given pathway mixed with genes that positively regulate that pathway. These “mixed” results complicate our ability to determine the net effect of the changes in gene transcription that are associated with a specific pathway. Based on transcriptomic data alone, it is difficult to disentangle these complex patterns. Empirical, functional studies will be required to determine the combined effects of these changes in gene expression on the activity status of a given pathway and ultimately its contributions to changes in cell behavior.

Similar changes in aSMCs and vSMCs after uremic serum exposure also included the positive enrichment of cellular stress response signaling pathways and cellular waste-disposal signaling pathways, such as ER Stress Response, Autophagy, Mitophagy, Lysosome and Apoptosis. However, we noticed that the cellular responses to ER stress were completely different in aSMCs and vSMCs. In aSMCs, the protein processing in the ER pathway was predicted to be transcriptomically downregulated after uremic serum exposure, while in vSMCs, the protein processing in the ER pathway was predicted to be positively enriched in response to uremic serum. Suppression of protein processing in the ER pathway is a hallmark of uremic toxicity, as cells halt global protein synthesis due to stress conditions caused by the accumulation of uremic toxins and disruption of ER homeostasis [25]. At the early stages of ER stress, activation of the UPR is a primary adaptive defense mechanism triggered rapidly after exposure to uremic serum [26]. In this study, we revealed that several pathways related to the UPR were predicted to be enriched in vSMCs after uremic serum exposure, including the ERAD pathway, Regulation of Response to ER Stress, Intracellular Protein Transport, cellular UPR and proteasomal protein catabolic process.

In this study, we also detected the transcriptomic downregulation of cell structure-related signaling pathways in vSMCs after uremic serum exposure, such as Cytoskeleton in Muscle Cells, Regulation of Actin Cytoskeleton, and Focal Adhesion and Integrin signaling pathways, which was similar to our observations in aSMCs [19]. Suppression of these signaling pathways can physically disrupt cellular mechanotransduction and tension-bearing complexes, leading to the loss of smooth muscle contractility and cell stability [27,28,29,30]. In addition, the transcriptomic downregulation of the TGF-β signaling pathway was also detected in vSMCs after uremic serum exposure. Normally, the TGF-β signaling pathway actively promotes and maintains the differentiation of VSMCs [31]. Therefore, the alterations in these signaling pathways may contribute to the phenotypic switch of vSMCs from contractile to synthetic status after uremic serum exposure, which we observed in a prior study [20].

A major change detected in vSMCs that was not detected in aSMCs was positive enrichment in the cellular response to hypoxia [19]. Uremic toxins, such as indoxyl sulfate and p-cresyl sulfate, can promote and aggravate hypoxia at cellular and systemic levels [32,33]. Uremic toxins increase oxygen consumption and decrease overall tissue oxygenation [34]. Uremic serum also triggers eryptosis (premature red blood cell death) and inhibits the bone marrow from producing erythropoietin, the hormone responsible for making red blood cells, which can cause anemic hypoxia [35]. In addition, certain uremic toxins interfere with hypoxia-inducible factors (HIFs), the proteins that allow cells to adapt to low oxygen [36]. Indeed, our current study demonstrated that uremic serum exposure not only resulted in downregulated HIF-1α but also upregulated prolyl hydroxylase domain (PHD). PHD is an enzyme that adds hydroxyl groups to the HIF-1α subunit, leading to ubiquitin-mediated destruction by the proteasome [37], which can dramatically impair a cell’s ability to adapt to hypoxic conditions. Although upregulation of PHD may suppress the hypoxia response pathway, confirmation of changes in relevant protein levels and enzymatic activity is required. Furthermore, to survive under short-term hypoxia, the cell temporarily halts energy-intensive processes, such as cell division, DNA Replication and protein synthesis, and enhances the mTOR signaling pathway, which have also been detected in this study.

This study was a continuation of our previous candidate gene-based study that largely utilized protein-level approaches. The transcriptomic data and bioinformatic analyses presented here provide the foundation for future studies at the protein level or in animal models. However, bulk RNA-seq analysis only provides an average readout of gene expression across all cells contained in the samples, which may mask the cell types that are the major producers of a particular transcript. The lack of experimental validation of the key findings in this transcriptomic study limits our ability to determine which changes in gene expression (and implied changes in related signaling pathways) may contribute to or result from the response to uremic conditions. Nevertheless, this study provides initial guidance for investigators to explore the consequences of changes in gene expression on specific signaling pathways and vSMCs behavior based on their research interests. It is also worth noting that uremic serum exposure duration in the current study was relatively short; therefore, these results may represent an early-stage response of vSMCs to uremic serum. Longer-duration uremic serum exposure studies should provide a more complete understanding of cellular responses to uremic conditions.

In summary, the current transcriptomic study identified several signaling pathways that may be altered in vSMCs after uremic serum exposure. Notable examples include downregulated genes associated with Cell Cycle, DNA Replication, Cytoskeleton in Muscle Cells and Regulation of Actin Cytoskeleton, which were similar to our observations in aSMCs. These changes may contribute to the cell phenotypic switch, such as cellular dedifferentiation, that we observed in both aSMCs and vSMCs under uremic conditions. This study also identified vein-specific signaling pathways that may be altered after uremic serum exposure, such as Protein Processing in ER, Integrated Stress Response signaling pathway, Mitophagy and Cellular Response to Hypoxia, which showed much more positive responses in vSMCs. These pathways may represent novel targets for vein-specific clinical symptoms observed in CKD/ESKD patients, such as AVF stenosis and thrombosis.

## 4. Materials and Methods

### 4.1. Cell Culture and RNA Isolation

The animal study protocol was approved by the University of North Carolina at Chapel Hill IACUC (Chapel Hill, NC, USA) (IACUC ID: 22069 on 28 April 2022). The pig was housed at the UNC-Chapel Hill animal facility, which aligns with IACUC and USDA regulations that prioritize social interaction and animal welfare. Porcine vSMCs were isolated from the jugular veins of a 4-month-old female Yorkshire pig (62 kg). The cells were cultured with growth medium (DMEM containing 5 mM glucose and 30% normal pig serum or 30% uremic pig serum plus 1% penicillin/streptomycin). Uremic pig serum (BUN 43 mg/dL and creatinine 9.5 mg/dL) was obtained via a procedure described previously and uremic toxin values were determined [20]. All experiments were performed on cells at passage 6. When cells reached 100% confluency, total RNA from 3 plates of each treatment (120 h normal porcine serum or uremic porcine serum exposure) was isolated using an RNeasy Plus Mini Kit (Qiagen, Germantown, MD, USA) for bulk RNA sequencing (Novogene, Sacramento, CA, USA).

### 4.2. Differential Expression Analysis and DEGs Definition

Differential expression results for uremic porcine serum-treated vSMCs (UPV) vs. normal porcine serum-treated vSMCs (NPV) were summarized as log2 fold change (log2FC; computed as log2(UPV/NPV)) with corresponding nominal *p*-values and Benjamini–Hochberg adjusted *p*-values (FDR). For the volcano plot and the reported numbers of DEGs, genes with padj ≤ 0.05 and |log2FC| ≥ 1 were classified as significantly upregulated or downregulated.

### 4.3. GO BP Enrichment Analysis (Up vs. Down)

GO ORA was performed in R using the clusterProfiler package (version 4.18.4). and the porcine annotation package (org.Ss.eg.db) (version 3.22.0). Genes with padj < 0.05 were separated into genes expressed more highly in UPV and genes expressed less highly in UPV. No additional absolute log2FC cutoff was applied. Rows with missing gene identifiers, log2FC values, or adjusted *p*-values were excluded, and one entry was retained per Entrez Gene identifier.

GO enrichment was performed separately for the BP enrichment analysis. Benjamini–Hochberg multiple-testing correction was applied, and Entrez Gene identifiers were converted to readable gene symbols. No custom background gene universe was supplied. For visualization, terms were ordered by adjusted *p*-value, and the top 15 terms in each expression-direction group were displayed. GeneRatio represents the proportion of genes in the corresponding input gene list associated with each GO term, dot size represents the number of genes assigned to the term (Count), and dot color represents the Benjamini–Hochberg-adjusted enrichment *p*-value.

### 4.4. KEGG Pathway Enrichment ORA and GSEA

KEGG pathway enrichment was evaluated using ORA and GSEA in R with the clusterProfiler package and the Sus scrofa KEGG organism code “ssc”.

For ORA, genes with padj < 0.05 were separated into genes expressed more highly in UPV (log2FC > 0) and genes expressed less highly in UPV (log2FC < 0). No additional absolute log2FC cutoff was applied. The two directional gene sets were analyzed separately using enriched KEGG with Benjamini–Hochberg multiple-testing correction. Pathways with adjusted enrichment *p*-values ≤ 0.05 were retained for visualization. GeneRatio represents the proportion of genes in the corresponding directional input list assigned to each pathway, dot size represents count, and dot color represents the adjusted enrichment *p*-value.

For GSEA, rows with missing Entrez Gene identifiers, log2FC values, or adjusted *p*-values were excluded, and one entry was retained per Entrez Gene identifier. Genes were ranked in decreasing order according to log2FC, calculated as log2(UPV/NPV), and analyzed using gseKEGG with Benjamini–Hochberg multiple-testing correction. Pathways with adjusted *p*-values ≤ 0.05 were retained, and the top 15 pathways in each enrichment direction were displayed.

In the GSEA dot plot, the *x*-axis represents each pathway set size divided by the largest set size among the retained pathways. Dot size also represents pathway set size, and dot color represents the adjusted enrichment *p*-value.

### 4.5. KEGG Pathway Map Visualization

The KEGG database (https://www.kegg.jp/) was accessed [on 6 March 2026] to retrieve the latest pathway maps and annotations. Selected pathways were visualized by projecting gene-level log_2_FC values onto KEGG pathway schematics using Pathview. In pathway maps, node fill color represents log2FC (UPV/NPV): red indicates higher expression in UPV, and green indicates lower expression in UPV (relative to NPV).

### 4.6. Direct Comparison of Uremic-Serum Responses Between vSMCs and aSMCs

To compare transcriptomic responses to uremic serum between vSMCs and aSMCs, a cell type × serum interaction analysis was performed using DESeq2. Gene-level uremic-serum effects were estimated as log2 fold changes for vSMCs (UPV vs. NPV) and aSMCs (UPA vs. NPA), and the interaction term was used to identify genes whose responses differed significantly between the two subtypes of cells. Interaction significance was assessed using Benjamini–Hochberg adjusted *p*-values (FDR). Pearson’s correlation was used to compare gene-level uremic-serum effect sizes between vSMCs and aSMCs. For pathway-level comparison, genes were ranked according to the signed cell type × serum interaction statistic and analyzed by KEGG GSEA. Positive enrichment scores indicate a more positive uremic-serum response in vSMCs relative to aSMCs, whereas negative scores indicate a more positive response in aSMCs.

## Data Availability

The bulk RNA-seq raw data have been deposited at GEO (GSE343584), which are publicly accessible now. The authors declare that all data supporting the findings of this study will be available from the corresponding authors upon request.

## Supplementary Materials

The following supporting information can be downloaded at: https://www.mdpi.com/article/10.3390/ijms27188148/s1.
