# Supplementary material for: Alterations of Gene Expression and Signaling Pathway Activity in Venous Smooth Muscle Cells After Uremic Serum Exposure
Source: Int J Mol Sci. 2026 Sep 13;27(18):8148. doi: 10.3390/ijms27188148 (PMC13607795; doi:10.3390/ijms27188148)
Supplement: Supplementary file 1 [file ijms-27-08148-s001.zip › ijms-4478913-supplementary.pdf]

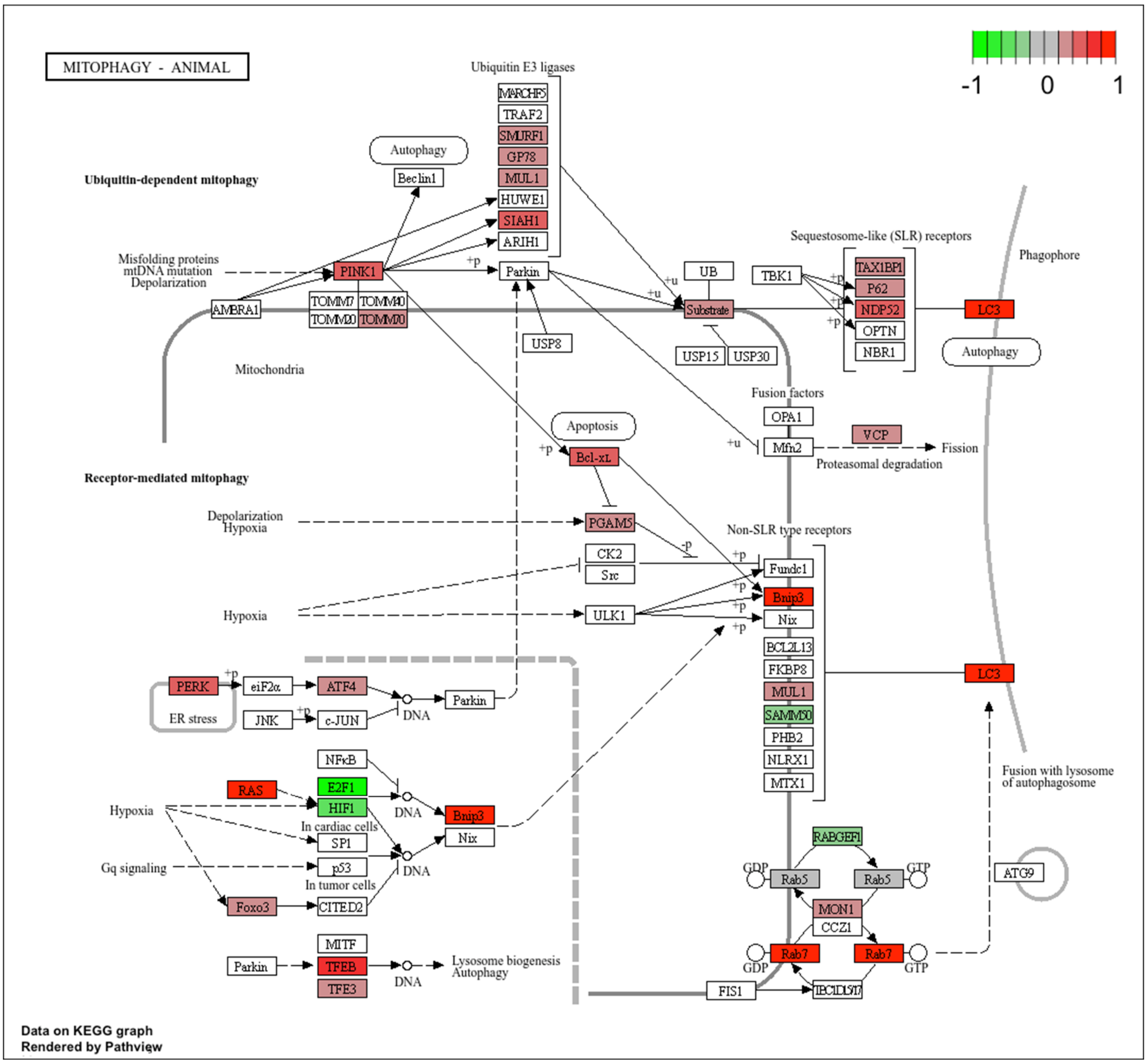


**Supplemental Figure S1. Mitophagy-animal pathway map in vSMCs exposed to uremic serum.** KEGG mitophagy-animal pathway with DEG log2FC values projected onto the pathway map. Node color represents log2FC (UPV/NPV), with red indicating upregulated expression in UPV and green indicating downregulated expression in UPV compared with NPV. This map highlights altered expression of genes involved in ubiquitin-dependent mitophagy and receptor-mediated mitophagy. The pathway structure is based on the KEGG pathway database (https://www.kegg.jp/kegg/pathway.html).

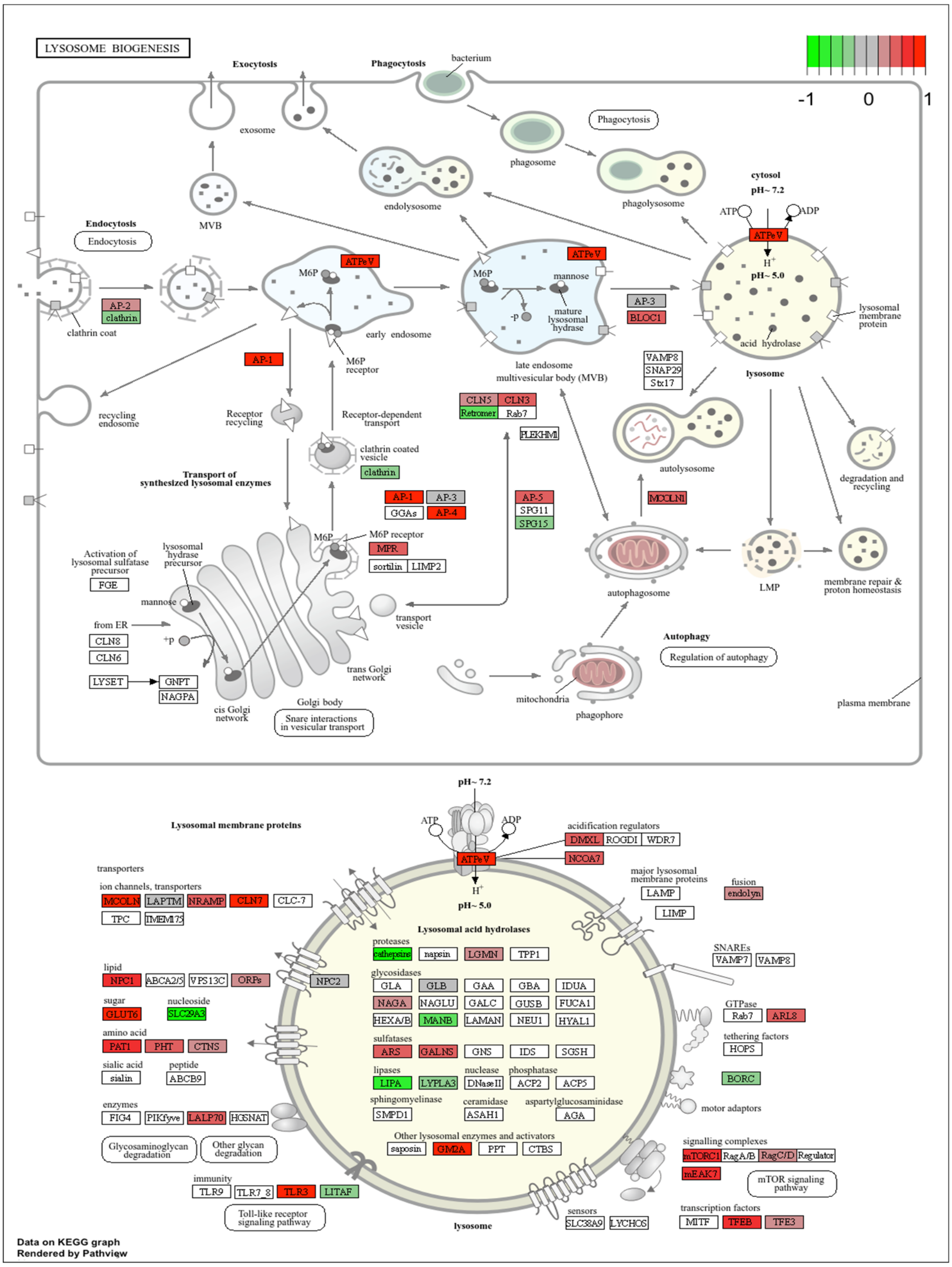

**Supplemental Figure S2. Lysosome Biogenesis pathway map in vSMCs exposed to uremic serum.** KEGG lysosome biogenesis pathway with DEG log2FC values projected onto the pathway map. Node color represents log2FC (UPV/NPV), with red indicating higher expression in UPV and green indicating lower expression in UPV compared with NPV. The pathway structure is based on the KEGG pathway database (https://www.kegg.jp/kegg/pathway.html).

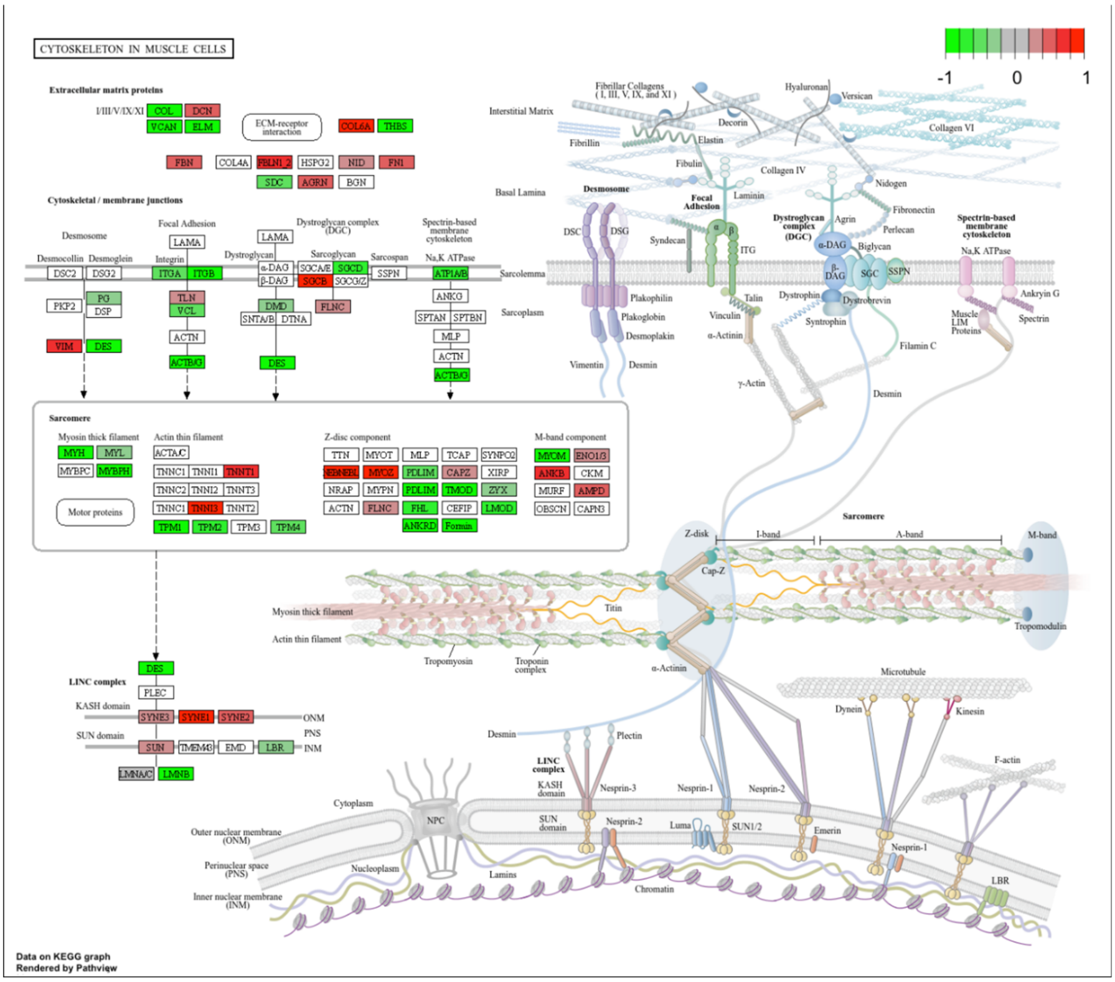


**Supplemental Figure S3. Cytoskeleton in Muscle Cells pathway map in vSMCs exposed to uremic serum.** KEGG cytoskeleton in muscle cells pathway with DEG log2FC values overlaid using Pathview. Red nodes indicate genes upregulated in UPV relative to NPV, and green nodes indicate genes downregulated in UPV. The pathway structure is based on the KEGG pathway database (https://www.kegg.jp/kegg/pathway.html).

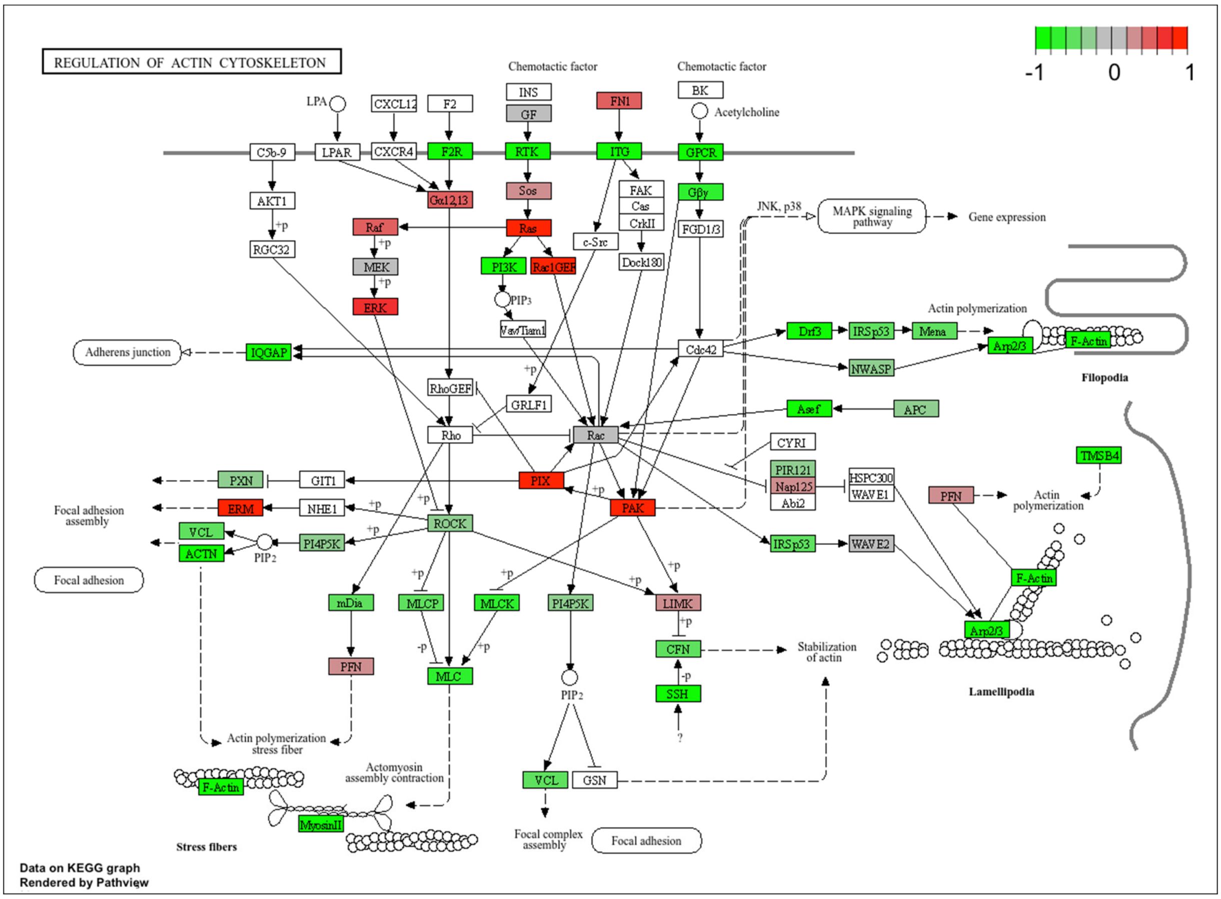


**Supplemental Figure S4. Regulation of Actin Cytoskeleton pathway map in vSMCs exposed to uremic serum.** KEGG regulation of actin cytoskeleton pathway with DEG log2FC values overlaid using Pathview. Red nodes indicate genes upregulated in UPV relative to NPV, and green nodes indicate genes downregulated in UPV. The pathway structure is based on the KEGG pathway database (https://www.kegg.jp/kegg/pathway.html).

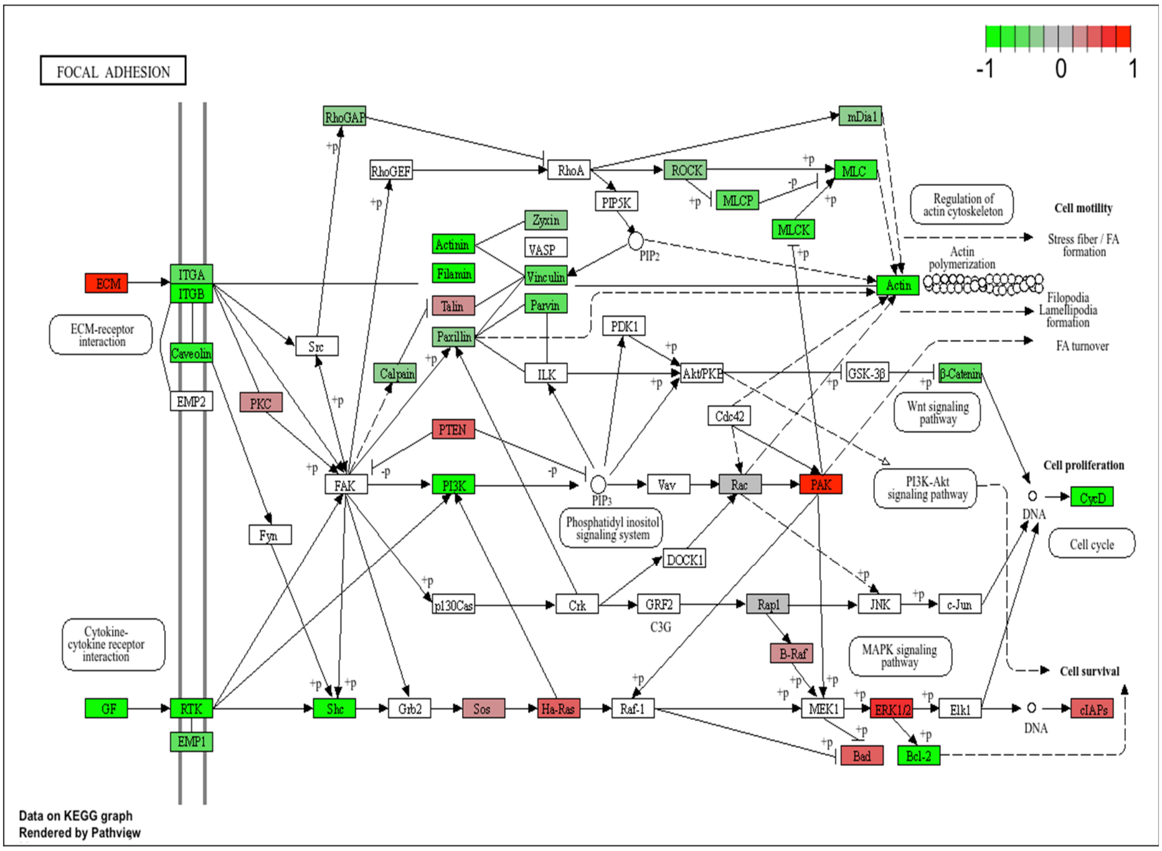


**Supplemental Figure S5. Focal Adhesion pathway map in vSMCs exposed to uremic serum.** KEGG focal adhesion pathway with DEG log2FC values projected onto the pathway map. Node color represents log2FC (UPV/NPV), with red indicating higher expression in UPV and green indicating lower expression in UPV compared with NPV. This map highlights altered expression of genes involved in extracellular matrix interaction, integrin signaling, cell proliferation, cell survival and cell motility. The pathway structure is based on the KEGG pathway database (https://www.kegg.jp/kegg/pathway.html).

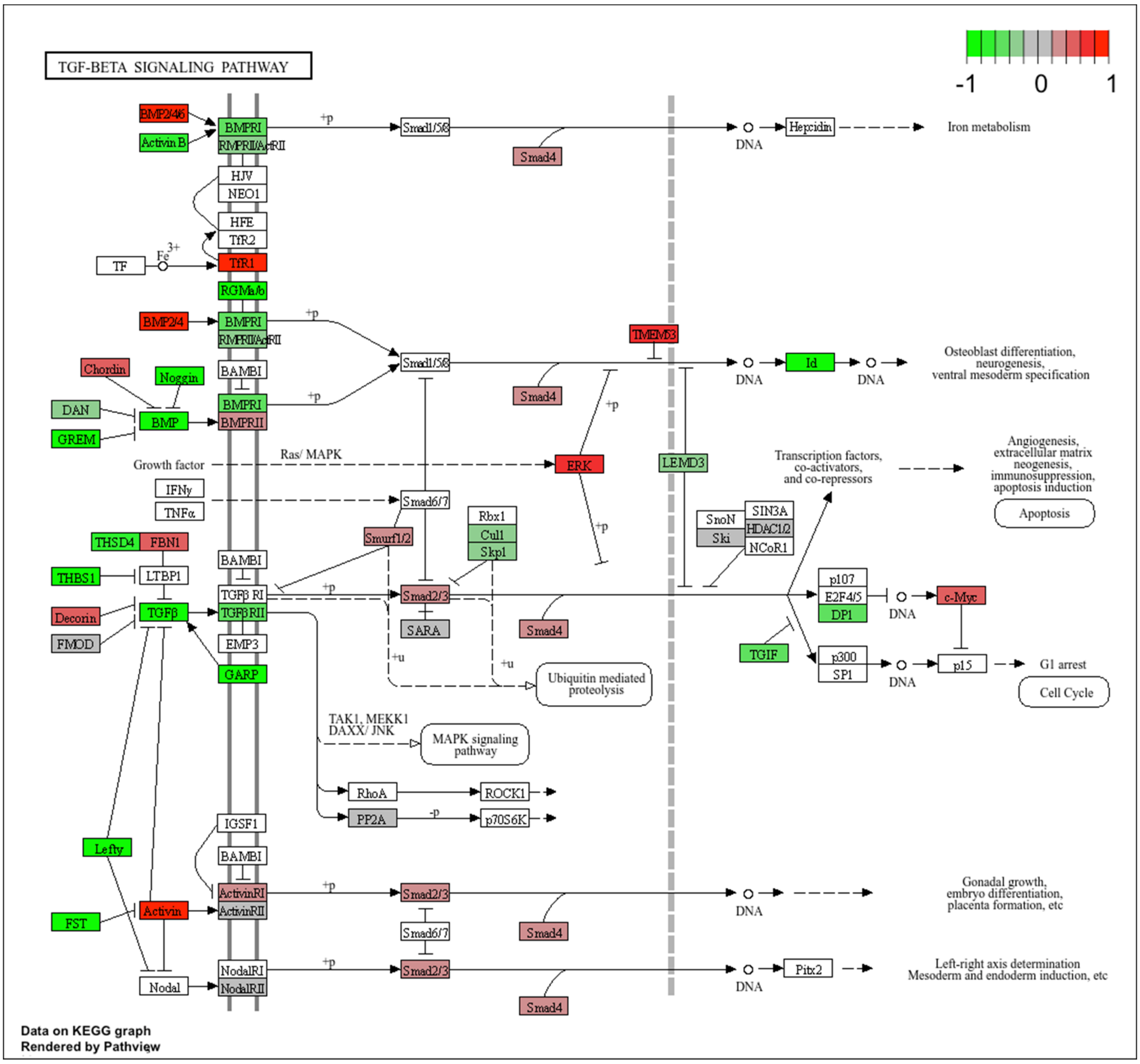


**Supplemental Figure S6. TGF-β signaling pathway map in venous smooth muscle cells exposed to uremic serum.** KEGG TGF-β signaling pathway with gene-level log2FC values overlaid using Pathview. Red indicates increased expression in UPV, and green indicates decreased expression in UPV relative to NPV. This pathway map highlights uremia-associated changes in TGF-β/BMP ligand signaling, receptor-associated signaling, SMAD-dependent transcriptional regulation, and downstream cell-cycle or extracellular matrix-related targets. The pathway structure is based on the KEGG pathway database (https://www.kegg.jp/kegg/pathway.html).

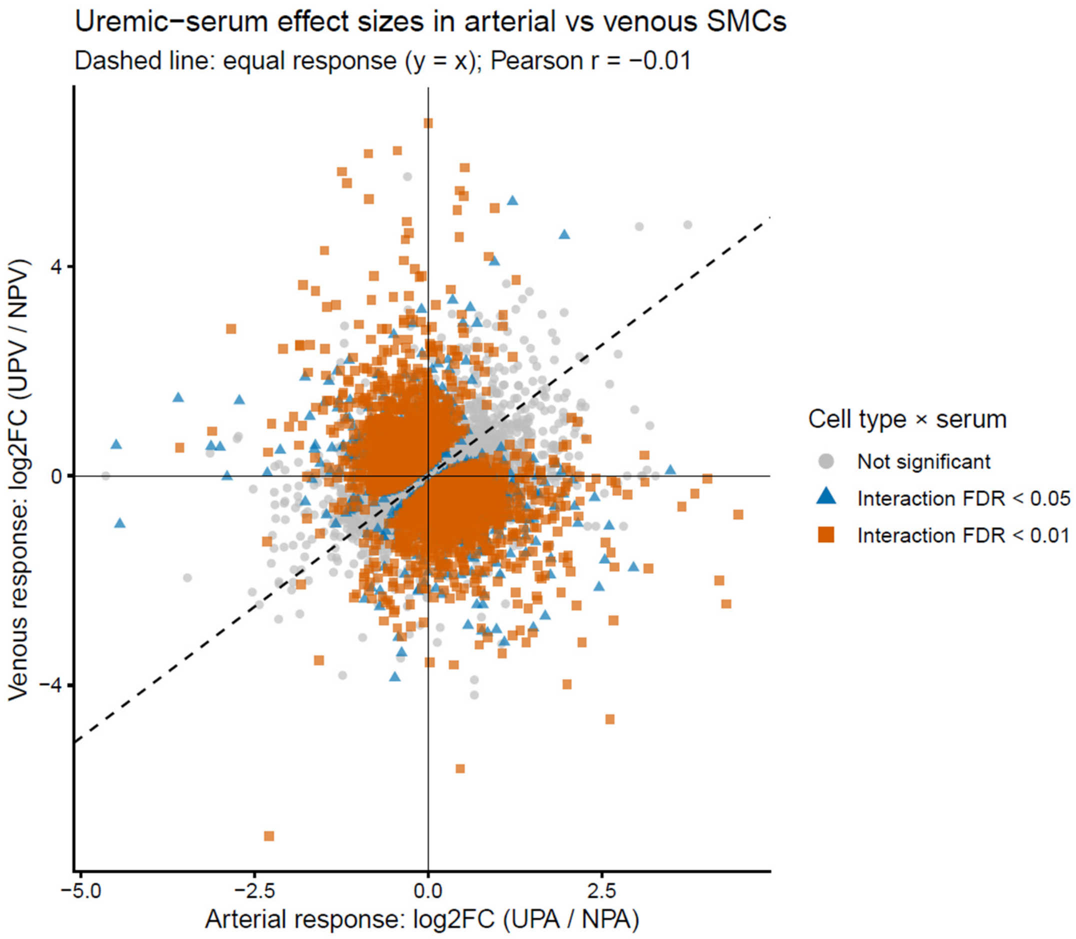


**Supplemental Figure S7. Comparison of uremic-serum response effect sizes between arterial and venous SMCs.** Gene-level responses to uremic serum were estimated using DESeq2 as log2 fold changes for arterial SMCs (UPA vs. NPA; x-axis) and venous SMCs (UPV vs. NPV; y-axis). A cell type × serum interaction model was used to test whether the uremic-serum response differed between arterial and venous SMCs. Gray circles represent genes without significant interaction, blue triangles indicate interaction FDR < 0.05, and orange squares show interaction FDR < 0.01. The dashed diagonal indicates equal uremic-serum responses in the two cell types (y = x). The low overall correlation (Pearson r = −0.01) indicates little concordance between arterial and venous gene-level responses, while genes displaced from the diagonal with significant interaction terms represent statistically different responses between the two cell types.

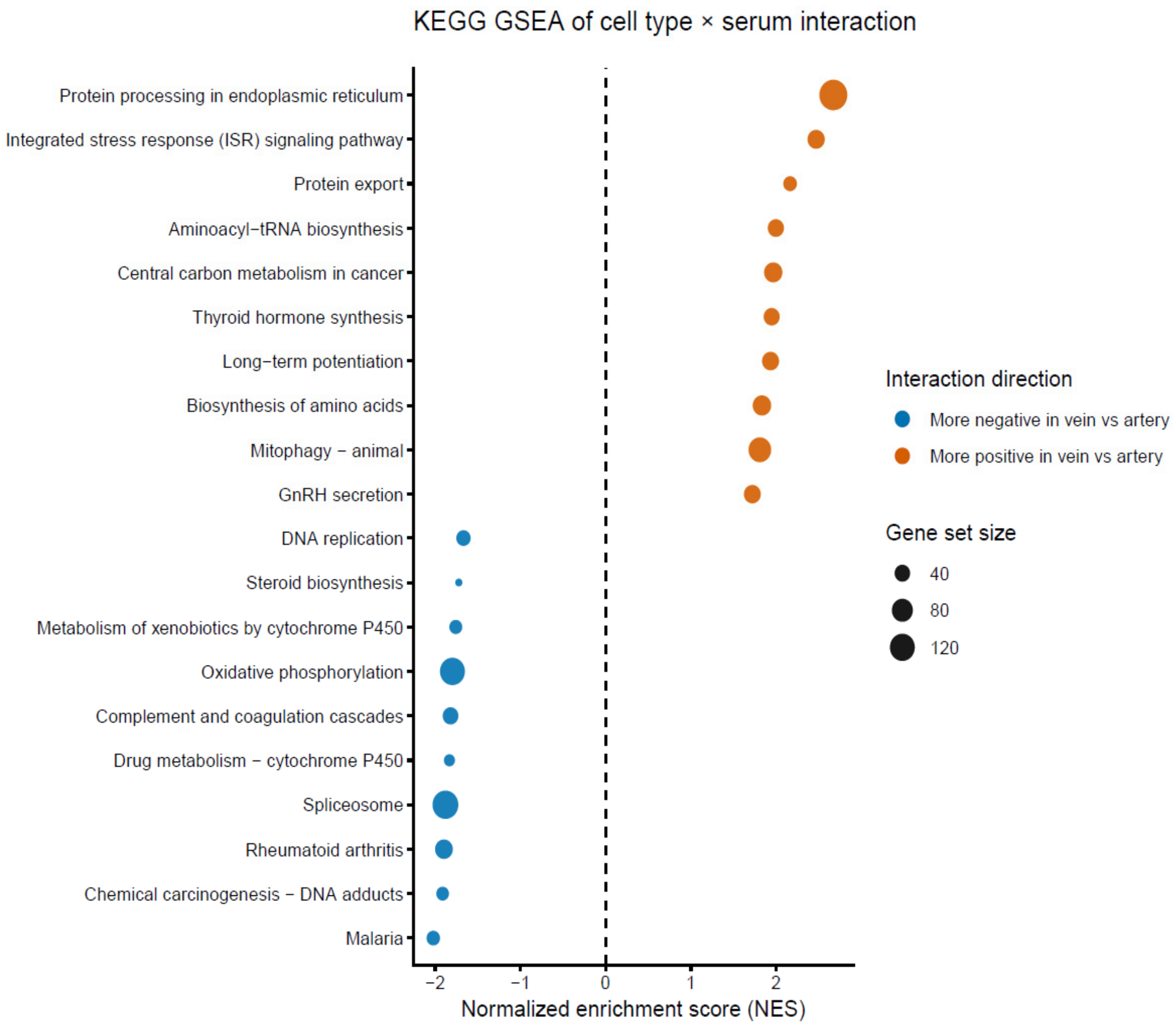


**Supplemental Figure S8. KEGG GSEA of the cell type × serum interaction.** Genes were ranked according to the signed cell type × serum interaction statistics and analyzed by KEGG Gene Set Enrichment Analysis (GSEA). The normalized enrichment score (NES) represents the direction and magnitude of the difference in pathway response between venous and arterial SMCs. Positive NES values indicate pathways whose response to uremic serum is shifted more positively in venous SMCs relative to arterial SMCs, whereas negative NES values indicate a more negative venous response. Point size represents gene-set size. Among the strongest positive interaction signals were protein processing in the endoplasmic reticulum and integrated stress response signaling, whereas oxidative phosphorylation and several other pathways showed negative interaction enrichment.
